# Design and evaluation of a novel fusion antigen for diagnosing human strongyloidiasis: An immunoinformatics approach

**DOI:** 10.1371/journal.pone.0351189

**Published:** 2026-06-25

**Authors:** Marzieh Asadi, Sina Taghvimi, Ghazal Ghaznavi, Halime Parsaee, Mehdi Mohsenzadeh, Bahador Sarkari, Mohammad Jafari, Amir Savardashtaki

**Affiliations:** 1 Cellular and Molecular Research Center, Gerash University of Medical Sciences, Gerash, Iran; 2 Department of Medical Biotechnology, School of Advanced Medical Sciences and Technologies, Shiraz University of Medical Sciences Shiraz, Shiraz, Iran; 3 Department of Biology, Faculty of Sciences, Shahid Chamran University of Ahvaz, Ahvaz, Iran; 4 Student Research Committee, Shiraz University of Medical Sciences, Shiraz, Iran; 5 Student Research Committee, Gerash University of Medical Sciences, Gerash, Iran; 6 Gerash Al-Zahra Fertility Center, Gerash University of Medical Sciences, Gerash, Iran; 7 Department of Parasitology and Mycology, School of Medicine, Shiraz University of Medical Sciences, Shiraz, Iran; 8 Infertility Research Center, Shiraz University of Medical Sciences, Shiraz, Iran; Islamic Azad University, IRAN, ISLAMIC REPUBLIC OF

## Abstract

Strongyloidiasis, caused by *Strongyloides stercoralis*, remains a neglected tropical disease (NTD) with significant clinical implications, particularly in immunocompromised individuals. Current serological assays for diagnosing strongyloidiasis are limited by suboptimal sensitivity and specificity. The development of recombinant fusion proteins for serodiagnostic applications represents a promising strategy to improve diagnostic accuracy. This study aimed to design a novel recombinant fusion antigen for the serodiagnosis of strongyloidiasis, using immunoinformatics approaches. Four immunogenic proteins (SsIR, L3NieAg.01, Ss3a, and Ss1a) were selected for the design of the fusion antigen. The most immunogenic regions of these proteins were identified based on epitope density and minimal cross-reactivity, and they were linked, using EAAAK linkers. The designed fusion antigen was then evaluated for its physicochemical properties, solubility, antigenicity, and potential cross-reactivity. Its three-dimensional (3D) structure was predicted, and the nucleotide sequence was codon-optimized to ensure efficient expression in *Escherichia coli* (*E. coli*). Finally, the optimized sequence was in silico cloned into the pET23a(+) expression vector. Immunoinformatics analyses demonstrated that the designed fusion antigen exhibits appropriate stability and robust antigenicity while showing no significant cross-reactivity. Codon optimization resulted in a codon adaptation index (CAI) of 0.92, and a GC content adjusted to 47%, confirming its compatibility with the *E. coli* expression system. Furthermore, no inhibitory cis-regulatory elements or repetitive sequences were identified post-optimization, supporting the feasibility of successful recombinant expression in *E. coli*. The bioinformatics findings of this study indicate that the designed fusion antigen holds significant potential for incorporation into ELISA-based serodiagnostic assays for strongyloidiasis.

## Introduction

*Strongyloides stercoralis* is a significant parasite that can cause chronic and severe infections. While strongyloidiasis is primarily caused by *S. stercoralis*, other species such as *S. fuelleborni* and *S. kellyi* can also cause this disease, although less frequently. Recognizing its importance, the World Health Organization (WHO) has, for the first time, included *S. stercoralis* as a target pathogen in its 2021–2030 neglected tropical diseases (NTD) control program [1].

*S. stercoralis* enters the human body through the penetration of the skin by its filariform larvae present in contaminated soil. Once inside, the larvae migrate through the bloodstream to the lungs, then reach the throat, where they are swallowed and eventually become adult worms in the small intestine. Unlike many other nematodes, the eggs of this parasite hatch within the host’s body, releasing rhabditiform larvae that are excreted in the stool. These larvae can either develop into free-living adults in the environment or transform back into infectious filariform larvae, continuing the cycle of infection [2]. One of the unique characteristics of this parasite is autoinfection. Instead of being excreted, some newly produced larvae directly re-enter the bloodstream through the intestinal wall or perianal skin. This allows the parasite to sustain the infection within the host’s body without external exposure. As a result, an individual can remain infected for years or even decades without re-exposure to a contaminated environment [3].

Under normal conditions, many infected individuals may remain asymptomatic or experience mild symptoms such as abdominal pain, intermittent diarrhea, and skin rashes. However, in immunocompromised patients, especially those receiving corticosteroid therapy, the risk of hyperinfection and disseminated disease increases significantly. Other predisposing factors include infection with *human T-lymphotropic virus type 1* (*HTLV-1*), organ transplantation, hematologic malignancies (particularly lymphoma), hypogammaglobulinemia, chronic alcohol consumption, kidney failure, severe malnutrition, diabetes, and chemotherapy [4]. In such cases, larvae spread uncontrollably throughout the body, invading organs such as the lungs, liver, brain, and genitourinary system. This condition can lead to life-threatening complications, including respiratory failure, sepsis, and septic shock. The mortality rate in these patients is extremely high and, if not diagnosed and treated in time, can exceed 70% [5].

The diagnosis of strongyloidiasis has traditionally relied on the direct observation of larvae in stool samples. However, single-sample microscopic methods have a low sensitivity of approximately 21%, primarily due to the intermittent shedding of the parasite and low infection burden (3). Polymerase chain reaction (PCR) also faces significant challenges among molecular methods. One of the main limitations of PCR is its reduced sensitivity caused by the intermittent excretion of the parasite, leading to false-negative results even in the presence of infection. Additionally, the requirement for meticulous sample processing, high costs, technical complexity, and limited accessibility in resource-poor settings are major barriers to the widespread use of this technique. Furthermore, despite PCR’s high efficacy in detecting active infections, it does not provide information about past infections or assess treatment effectiveness [6]. Serological methods, particularly enzyme-linked immunosorbent assay (ELISA), are widely used for detecting infectious agents due to their high speed, low cost, and reproducibility. However, the use of native antigens in these assays presents several challenges. The extraction and production of these antigens are complex and costly processes that can introduce impurities, leading to false-positive results. Additionally, cross-reactivity remains a fundamental issue affecting diagnostic accuracy [7].

To enhance the precision of serological methods, the design of recombinant antigens using bioinformatics approaches has emerged as a novel and promising strategy. By integrating multiple sequences from the target parasite and focusing on highly immunogenic regions, these antigens enable broader antibody detection while reducing the likelihood of false-positive results due to cross-reactivity [8]. Research on infectious disease diagnostics using ELISA has demonstrated that employing multi-epitope and fusion antigens can significantly improve both the sensitivity and specificity of the assay. These advancements play a crucial role in enhancing the accuracy and efficiency of serological diagnostic methods [9,10].

In this study, four excretory–secretory (ES) proteins of Strongyloides stercoralis SsIR, L3NieAg.01, Ss1a, and Ss3a were selected as candidates for recombinant antigen design. These proteins were chosen based on previous evidence demonstrating their strong immunogenicity, consistent expression during clinically relevant life‑cycle stages, and proven recognition by host antibodies in infected individuals. Collectively, these characteristics make them suitable components for constructing an effective fusion antigen [11,12].

This study aimed to design a novel recombinant fusion antigen for the serodiagnosis of strongyloidiasis using immunoinformatics approaches. The anticipated outcome of this research is the identification of a promising antigen candidate with significant potential for incorporation into ELISA-based serodiagnostic assays for strongyloidiasis. The bioinformatics findings generated in this study are expected to facilitate the development of more accurate, sensitive, and specific diagnostic tools, ultimately contributing to improved detection and control of this neglected parasitic infection.

## Methods

### Study design

**Fig 1:**
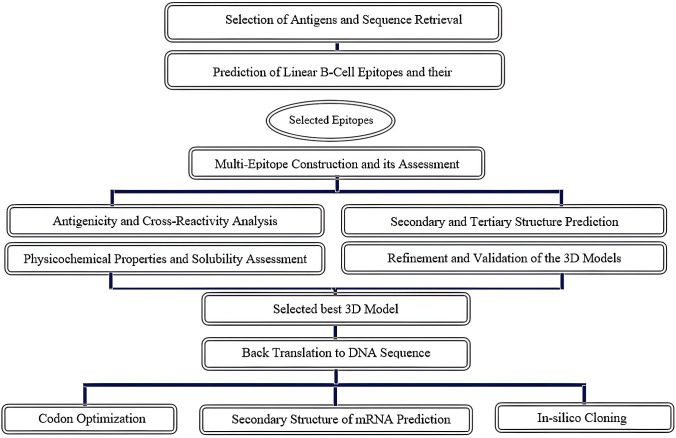
Flowchart illustrated a fusion antigen’s in-silico design stages for diagnosing strongyloidiasis.

The overall workflow of this study was shown in Fig 1, which provided a summary of the methodological steps. Four immunogenic proteins (SsIR, L3NieAg.01, Ss3a, and Ss1a) were selected for the design of the fusion antigen. The most immunogenic regions of these proteins were identified based on epitope density and minimal cross-reactivity, and they were linked, using EAAAK linkers. The designed fusion antigen was then evaluated for its physicochemical properties, solubility, antigenicity, and potential cross-reactivity. Its three-dimensional (3D) structure was predicted, and the nucleotide sequence was codon-optimized to ensure efficient expression in *Escherichia coli* (*E. coli*). Finally, the optimized sequence was in silico cloned into the pET23a(+) expression vector.

### Antigen selection and sequence retrieval

A comprehensive literature review was conducted to identify four antigenic sequences based on their immunogenic properties as reported in previous experimental studies. Selection criteria included evidence of immunoreactivity in ELISA or immunoblot assays and the potential for diagnostic application. The selected antigens included SsIR (GenBank ID: AAB97359.1, UniProt ID: O44394) and L3NieAg.01 (GenBank ID: AAD46493.1, UniProt ID: Q9UA16), as well as Ss3a and Ss1a (Patent No: WO2017091059A1) [13]. The amino acid sequences were retrieved from the UniProt database (https://www.uniprot.org/) in FASTA format, as summarized in Table 1.

**Table 1:** Selected antigenic proteins for the diagnosis of strongyloidiasis infection.

GenBank ID	UniProt ID	Patent ID	Status	Protein name	Amino acids length (aa)	Location
AAB97359.1	O44394	–	Un-reviewed	• IgG immunoreactive antigen • Immunoreactive antigen SsIR	156	Excretory-Secretory
AAD46493.1	Q9UA16	–	Un-reviewed	L3NieAg.01	229	Excretory-Secretory
–	**–**	WO2017091059A1	–	Ss3a	224	Excretory-Secretory
–	**–**	WO2017091059A1	–	Ss1a	297	Excretory-Secretory

### Antigenicity and cross-reactivity analysis

The antigenicity of each sequence was evaluated using the VaxiJen v2.0 server (http://www.ddg-pharmfac.net/vaxijen/VaxiJen/VaxiJen.html) with a threshold value set at 0.5. The prediction accuracy of this server varies between 70% and 89%, depending on the target organism [14]. VaxiJen, it is an alignment-independent server designed to predict protective antigenicity of proteins based solely on their physicochemical properties rather than sequence similarity. It applies an auto cross-covariance (ACC) transformation, which converts amino acid sequences into uniform numerical vectors by quantifying properties such as hydrophobicity, molecular size, and polarity. These vectors are then classified using machine learning models trained on datasets of known antigens and non-antigens. VaxiJen thus enables the identification of potential antigenic proteins, including those that may not show significant sequence similarity to known antigens.

Pathogenic organisms or closely related species that may share similar epitopes with the target sequences were identified. Cross-reactivity was assessed against a panel of parasitic infections of clinical significance to humans, including toxocariasis, fascioliasis, hydatidosis, malaria, hymenolepiasis, visceral leishmaniasis, toxoplasmosis, cryptosporidiosis, giardiasis, trichostrongylosis, and hookworm infections. The potential for cross-reactivity of each antigen (SsIR, L3NieAg.01, Ss3a, Ss1a) was evaluated using the BLASTp tool (https://blast.ncbi.nlm.nih.gov/Blast.cgi).

### Prediction of signal peptide

SignalP 6.0 (https://services.healthtech.dtu.dk/services/SignalP-6.0/) is specifically designed and optimized for the accurate detection and characterization of signal peptides across a diverse range of organisms, including archaea, gram-positive bacteria, gram-negative bacteria, and eukaryotes. utilizing advanced deep learning algorithms, this server ensures high-precision predictions, enabling the reliable identification of signal peptides and their cleavage sites [15].

### Prediction of linear B-cell epitopes for identifying antigenic regions

Three computational servers were utilized to predict linear B-cell epitopes within each antigen sequence. The Bcpred server (https://webs.iiitd.edu.in/raghava/bcepred/bcepred%20submission.html) predicts linear B-cell epitopes by analyzing various physicochemical properties of amino acids [16]. The ABCpred server (https://webs.iiitd.edu.in/raghava/abcpred/ABC_submission.html) employs an artificial neural network algorithm to analyze protein sequences and identify possible epitope regions [17,18]. The BepiPred server (https://services.healthtech.dtu.dk/service.php?BepiPred-2.0) utilizes a machine learning algorithm that integrates multiple physicochemical properties of amino acids to estimate the probability of a given region functioning as a linear B-cell epitope [19].

### Fusion antigen construction

The fusion antigen was designed by selecting specific regions from each antigen sequence based on two key criteria: minimizing cross-reactivity and maximizing epitope density. These selected regions were then linked using the EAAAK amino acid linker.

### Antigenicity, cross-reactivity, and physicochemical properties

The antigenicity of the designed fusion antigen was predicted using the VaxiJen v2.0 server with a threshold value of 0.5. A BLASTp analysis was performed to evaluate potential cross-reactivity. Additionally, the physicochemical properties of the fusion antigen were assessed using the ProtParam tool, available at (https://www.expasy.org/tools/protparam.html(. This tool provides essential data on various physicochemical characteristics, including amino acid composition, theoretical isoelectric point (pI), molecular weight (Mw), instability index, and aliphatic index [20]. Furthermore, the solubility of the fusion protein was predicted using the SolPro server (https://scratch.proteomics.ics.uci.edu/), which employs a machine-learning approach to estimate protein solubility. This prediction is based on multiple parameters such as amino acid composition, hydrophobicity, secondary structure, solvent accessibility, and overall charge [21,22].

### Prediction of secondary and tertiary structures and validation of the 3D model

The secondary and tertiary structures of the designed fusion antigen were predicted using the PSIPRED and GalaxyTBM servers, respectively. PSIPRED (http://bioinf.cs.ucl.ac.uk/psipred/) employs a combination of neural networks and hidden Markov models to provide highly accurate predictions of the protein’s secondary structure. Based on the input amino acid sequence, this server categorizes the protein structure into three significant elements: alpha-helices, beta-strands, and coils [23].

The tertiary structure of the designed fusion antigen was predicted using the GalaxyTBM server (https://galaxy.seoklab.org/cgi-bin/submit.cgi?type=TBM). This server employs Template-Based Modeling (TBM) to generate five structural models of the antigen [24,25]. The predicted tertiary structures were visualized using the PyMOL software. To ensure the accuracy and reliability of the predicted models, all generated structures were evaluated using the PROCHECK server (https://saves.mbi.ucla.edu/). This server assesses the quality of the protein structure by analyzing the Ramachandran plot, which examines the dihedral angles (ψ and φ) of amino acid residues to validate the geometric integrity of the protein structure [26].

### Prediction of conformational B-cell epitopes

Prediction of conformational B-cell epitopes was performed using the Ellipro server (http://tools.iedb.org/ellipro/). This server employs an algorithm integrating three critical structural parameters: solvent accessibility, spatial clustering, and surface propensity to identify potential epitope regions on the antigen’s 3D structure. The prediction analysis used default parameters, with a minimum score threshold of 0.5 and a maximum inter-residue distance cutoff of 6 Å [27].

### Codon optimization and rare codon analysis

The amino acid sequence of the designed fusion antigen was back-translated into a nucleotide sequence using the EMBOSS server (https://www.ebi.ac.uk/jdispatcher). To enhance the expression efficiency of the target protein in the *E. coli* host, the sequence was optimized, and its quality was evaluated based on key parameters, including the codon adaptation index (CAI), frequency of optimal codons (FOP), and GC content, using Gene Universal (USA). Additionally, rare codon analysis was performed using the online tool at https://www.genscript.com/tools/rare-codon-analysis. This analysis aimed to identify the presence of CIS and repeat elements within the sequence, potentially hindering protein expression.

### Prediction of secondary structure of mRNA

To predict the secondary structure of the mRNA molecule, the optimized DNA sequence was first transcribed into its corresponding RNA sequence using the DNA > RNA > Protein tool (https://biomodel.uah.es/en/lab/cybertory/analysis/trans.htm). Subsequently, the secondary structure of the mRNA was predicted using the mfold server (http://www.unafold.org/mfold/applications/rna-folding-form.php) [28].

### Recombinant vector design

For recombinant vector design, SnapGene v5.1.4.1 software was utilized to insert the fusion antigen sequence into the multiple cloning site (MCS) of the pET-23a (+) expression vector at the *NdeI* and *XhoI* restriction sites.

## Results

### Prediction of signal peptide

**Fig 2:**
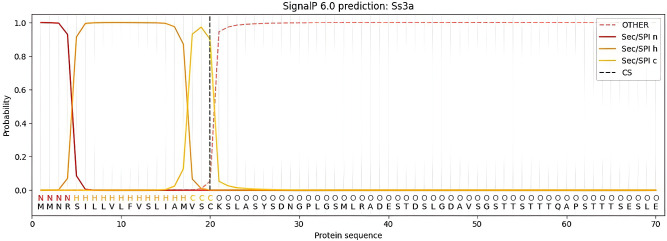
Signal peptide in Ss3a antigen, with the black dashed line indicating the cleavage site (~position 20).

Signal peptide prediction was performed using the SignalP server (Fig 2). Among the analyzed antigens (SsIR, L3NieAg.01, Ss3a, and Ss1a), a signal peptide was predicted only for the Ss3a antigen at its N‑terminal region. This signal peptide consisted of three distinct segments: a positively charged region (residues 1–5, shown in red), a hydrophobic region (residues 6–17, shown in orange), and a cleavage region (residues 18–20, shown in yellow). The predicted cleavage site (CS), indicated by a black dashed line, was located around position 20, suggesting the position where the signal peptide is removed to generate the mature protein.

### Prediction of linear B-cell epitopes

**Fig 3:**
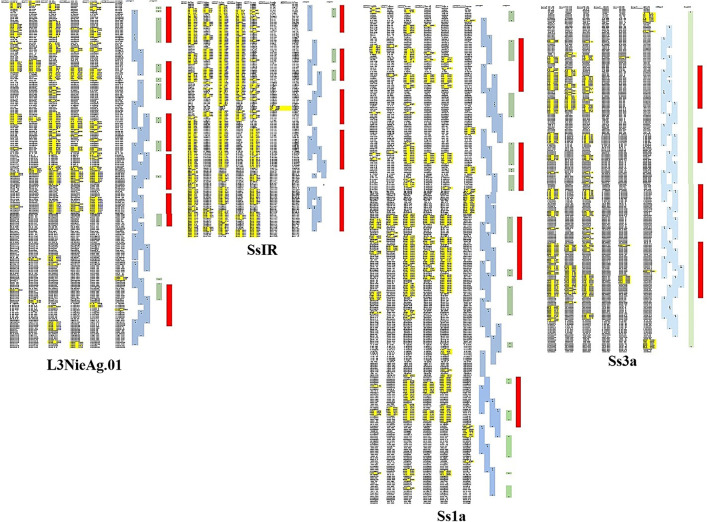
Predicted linear B-cell epitopes in four antigenic proteins using BCPRED (yellow), ABCpred (blue bars), and BepiPred (green bars). Red bars indicate consensus regions identified by all three servers.

Linear B‑cell epitopes in the four antigenic proteins were predicted using the BCPRED, ABCpred, and BepiPred servers. The predicted epitope regions are shown in Fig 3. In the protein sequences, epitopes identified by BCPRED, representing the highest‑scoring regions, are highlighted in yellow. Epitopes predicted by ABCpred are indicated by blue bars, whereas those identified by BepiPred are shown with green bars. Regions predicted as epitopes by all three servers are highlighted in red.

### Construction of the fusion antigen

**Fig 4:**
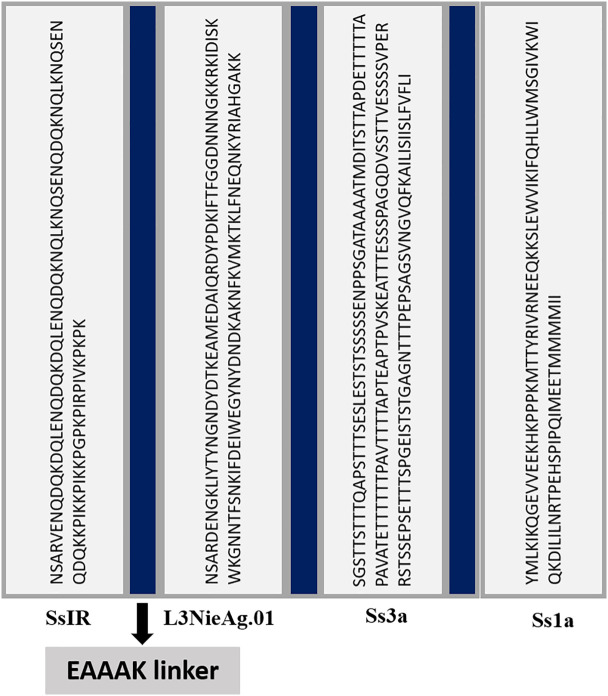
Schematic representation of the fusion antigen, in which the selected regions (SsIR, L3NieAg.01, ss3a, ss1a) are linked using the EAAAK linker.

The fusion antigen was designed by selecting protein regions with a high density of predicted epitopes and no detectable cross‑reactivity. The selected regions were SsIR (residues 1–79), L3NieAg.01 (residues 1–107), Ss3a (residues 50–124), and Ss1a (residues 213–297). These fragments were joined using the (EAAAK) amino acid linker to construct the fusion antigen. The final structure of the designed fusion antigen is shown in Fig 4.

### Antigenicity and cross-reactivity analysis

The predicted cross‑reactivity profiles of the full‑length antigens, their selected truncated regions, and the final fusion antigen were evaluated using BLASTp against proteins from several parasitic pathogens commonly associated with serological cross‑reactions. As shown in Table 2, the full‑length antigens exhibited varying degrees of sequence similarity with proteins from other parasites. Among them, SsIR demonstrated the highest level of potential cross‑reactivity, showing similarity with proteins from eight infections, including *Toxocara*, *Echinococcus*, *Plasmodium*, *Hymenolepis*, *Leishmania*, *Cryptosporidium*, *Trichostrongylus*, and *Necator americanus*. The L3NieAg.01 antigen also showed predicted similarity with seven infections, including *Toxocara*, *Fasciola*, *Echinococcus*, *Hymenolepis*, *Toxoplasma*, *Trichostrongylus*, and *Necator americanus*. In contrast, Ss3a did not show detectable similarity with any of the evaluated parasites, suggesting a potentially higher level of specificity. The Ss1a antigen displayed limited cross‑reactivity, with sequence similarity observed only with *Toxocara*, *Plasmodium*, and *Cryptosporidium*. Importantly, none of the selected truncated fragments used for constructing the fusion antigen showed detectable similarity with the evaluated parasitic pathogens in the BLASTp analysis. Consequently, the final fusion antigen also showed no predicted cross‑reactivity with any of the investigated parasites (Table 2).

**Table 2:** Predicted cross‑reactivity of full‑length antigens, selected truncated regions used in this study, and the designed fusion antigen with various parasitic pathogens based on BLASTp homology analysis.

Infections	Causative agent	Taxid	Cross-reactivity								
			Full antigen				Truncate antigen [In this study]				Fusion antigen [In this study]
			SsIR	L3NieAg.01	Ss3a	Ss1a	SsIR (1-79)	L3NieAg.01 (1-107)	Ss3a (50-224)	Ss1a (213-297)	
Toxocariasis	*Toxocara*	6264	Yes	Yes	No	Yes	No	No	No	No	No
Fascioliasis	*Fasciola*	6191	No	Yes	No	No	No	No	No	No	No
Hydatidosis	*Echinococcus*	6209	Yes	Yes	No	No	No	No	No	No	No
Malaria	*Plasmodium*	5820	Yes	No	No	Yes	No	No	No	No	No
Hymenolepiasis	*Hymenolepis*	6215	Yes	Yes	No	No	No	No	No	No	No
Visceral leishmaniasis	*Leishmania*	5658	Yes	No	No	No	No	No	No	No	No
Toxoplasmosis	*Toxoplasma gondii*	5811	No	Yes	No	No	No	No	No	No	No
Cryptosporidiosis	*Cryptosporidium*	5806	Yes	No	No	Yes	No	No	No	No	No
Giardiasis	*Giardia lamblia*	5741	No	No	No	No	No	No	No	No	No
Trichostrongylosis	*Trichostrongylus*	6318	Yes	Yes	No	No	No	No	No	No	No
Hookworm infection	*Ancylostoma Duodenale*	51022	Yes	Yes	No	No	No	No	No	No	No
	*Ancylostoma ceylanicum*	53326	No	Yes	No	No	No	No	No	No	No
	*Necator americanus*	51031	Yes	Yes	No	No	No	No	No	No	No

The antigenicity of the full‑length antigens, the selected truncated regions used in this study, and the final fusion construct was evaluated using the VaxiJen server with a threshold of 0.5. As summarized in Table 3, all analyzed sequences were predicted to be antigenic. Among the full‑length antigens, SsIR showed the highest antigenicity score (0.8672), followed by Ss3a (0.8616), L3NieAg.01 (0.6152), and Ss1a (0.5116). Notably, the selected fragment of SsIR (residues 1–79) exhibited the highest predicted antigenicity score (0.9873). The selected fragments from L3NieAg.01, Ss3a, and Ss1a also maintained antigenicity scores above the defined threshold. The designed fusion antigen showed a VaxiJen score of 0.6949, indicating that the antigenic potential was preserved after combining the selected fragments.

**Table 3:** Predicted antigenicity scores of full-length antigens, selected truncated regions, and the final designed fusion antigen.

Antigenicity*								
Full antigen				Truncate antigen [In this study]				Fusion antigen [In this study]
SsIR	L3NieAg.01	Ss3a	Ss1a	SsIR (1-79)	L3NieAg.01 (1-107)	Ss3a (50-224)	Ss1a (213-297)	
0.8672 Antigen	0.6152 Antigen	0.8616 Antigen	0.5116 Antigen	0.9873 Antigen	0.5421 Antigen	0.7654 Antigen	0.4756 Antigen	0.6949 Antigen

*Antigenicity was predicted using a threshold of 0.5.

### Physicochemical properties

The physicochemical properties of the fusion antigen were analyzed using the ProtParam server. The results indicated that the protein is stable, with an instability index of 39.68. The molecular weight was estimated to be approximately 50.81 kDa, and the predicted isoelectric point (pI) was 7.12. The aliphatic index was calculated as 57.83. In addition, SolPro analysis predicted that the fusion antigen is soluble, with a solubility score of 0.814387 (Table 4).

**Table 4:** Evaluation of a fusion antigen’s physicochemical properties, antigenicity, and cross-reactivity.

Physicochemical Properties						
Solubility#	Amino Acid Length	MW (kDa)	pI	Extinction coefficients (M ⁻ ¹·cm ⁻ ¹)	Aliphatic index	Stability Index*
0.814387 (Soluble)	461	50.81	7.12	40910	57.83	39.68 (Stable)

**Abbreviation:** Molecular Weight (MW); Isoelectric Point (pI).

# Proteins with solubility scores above 0.5 are considered soluble.

* Instability Index values below 40 indicate a stable protein.

### Prediction of secondary and tertiary structures and validation of the 3D model

**Fig 5:**
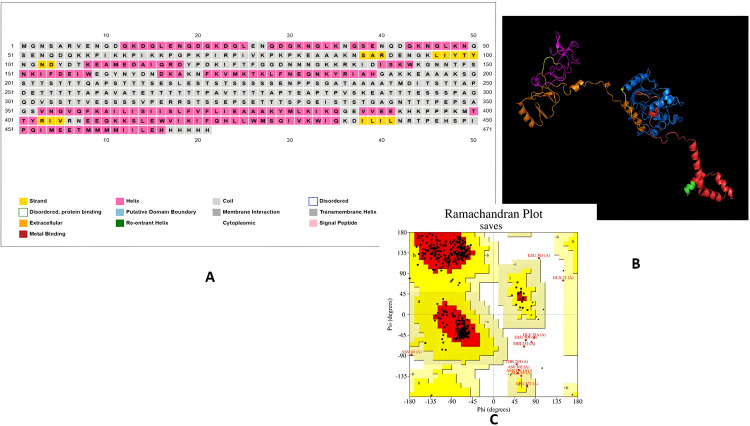
Prediction and evaluation of the fusion antigen structure. (a) Prediction of the secondary structure using the PSIPRED server. (b) The optimal 3D structural model generated by the GalaxyTBM server. (c) Ramachandran plot for structural quality assessment shows that 92.8% of the residues fall within the allowed regions.

The secondary structure of the fusion antigen was predicted using the PSIPRED server (Fig 5a). Tertiary structure prediction was performed with the GalaxyTBM server, which generated five candidate models. These models were assessed using Ramachandran plot analysis, and model 2 was selected as the optimal 3D structure (Fig 5b). In the selected model, 92.8% of the residues were located within the allowed regions (Fig 5c).

### Prediction of conformational B-cell epitopes

**Fig 6:**
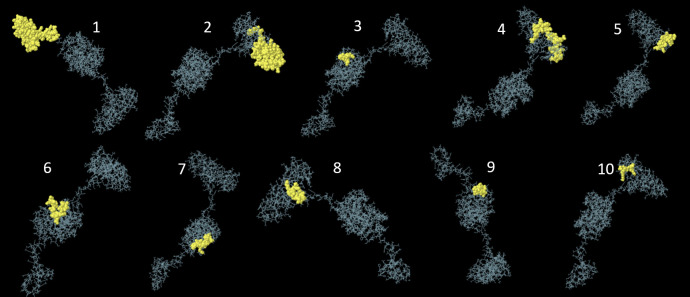
Prediction of conformational B-cell epitopes using the ElliPro server. The fusion antigen construct is represented as gray sticks, with the predicted conformational B-cell epitopes highlighted in yellow.

Conformational B-cell epitopes were predicted using the ElliPro server, which identified ten discontinuous epitopes comprising a total of 176 residues. The prediction scores ranged from 0.843 to 0.503, reflecting different confidence levels. The predicted epitopes varied in size from 6 to 66 amino acids. Detailed results are summarized in Table 5 and visualized in Fig 6.

**Table 5:** List of discontinuous B-cell epitopes in fusion antigen predicted by the ElliPro server.

No	Residues	Number of residues	Score
1	RNEEQKKSLEWVIKIFQHLLWMSGIVKWIQKDILILNRTPEHSPIPQIMEETMMMMIILEHHHHHH	66	0.843
2	KEAAAKNSARDENGKLIYTYNGNDYDTKEAMEDAIQRDYPDKIFTFGGDNNNGKKRKIDISKWKGNN	67	0.764
3	SLFVFL	6	0.697
4	MGNSARVENQDQKDQLENQDQKDQLENQDQKNQ	33	0.668
5	IKKPIKKPGPKPIRPIV	17	0.668
6	APSTTTSESLESTSTSS	17	0.66
7	VPERRSTSSEPSETT	15	0.604
8	ENQDQKNQ	8	0.593
9	STSTGAGNTTT	11	0.524
10	ENQDQK	6	0.503

### Quality assessment of the optimized sequence and prediction of mRNA secondary structure

**Fig 7:**
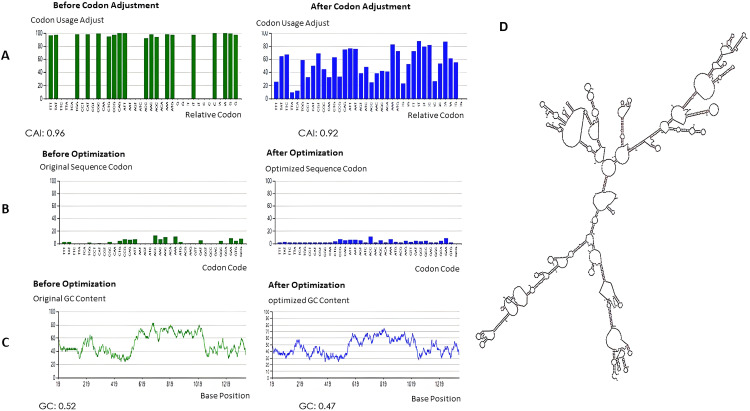
Evaluation of the optimized sequence and mRNA secondary structure for the designed fusion antigen. (a) Codon adaptation index (CAI) before (0.96) and after (0.92) codon adjustment. (b) Frequency of optimal codons (FOP) before and after optimization. (c) GC content before (52%) and after (47%) optimization. (d) Predicted mRNA secondary structure with a minimum free energy (MFE) −304.12 [Initially −391.00] kcal/mol.

Codon optimization was performed to improve the expression of the fusion antigen in *Escherichia coli*. After optimization, the codon adaptation index (CAI) of the original sequence decreased from 0.96 to 0.92, which remains within the recommended range for efficient expression (Fig 7a). The frequency of optimal codons (FOP) analysis showed that the original sequence contained a lower proportion of preferred codons, whereas the optimized sequence exhibited a substantial increase in the use of frequently employed codons, supporting improved translational efficiency (Fig 7b). The GC content was also adjusted to fall within the optimal range, decreasing from 52% to 47% (Fig 7c). The initial sequence contained one CIS element and six repetitive sequences, which were successfully eliminated following codon optimization (Table 6).

**Table 6:** Evaluation of negative CIS elements and repetitive sequences before and after codon optimization.

Sequence	Negative CIS elements	Negative repeat elements
None-optimized	1	6
Optimized	0	0

Furthermore, prediction of the mRNA secondary structure indicated that the minimum free energy (MFE) of the optimized sequence was −304.12 kcal/mol, a decrease from the original sequence’s MFE of −391.00 kcal/mol (Fig 7d).

### Recombinant vector design

**Fig 8:**
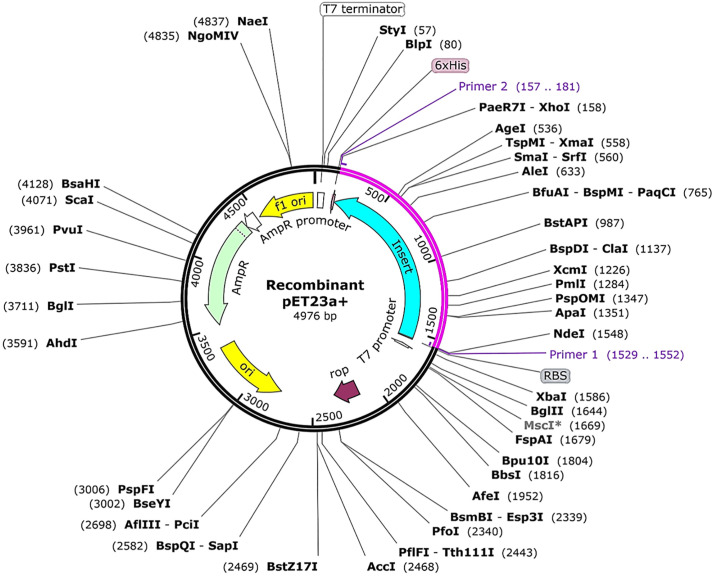
In-silico design of the recombinant pET-23a(+) vector containing the fusion antigen. The codon-optimized sequence (highlighted in pink) was inserted between the *NdeI* and *XhoI* restriction sites within the pET-23a(+) expression vector, representing a black circular structure.

The recombinant pET23a(+) vector, harboring the fusion antigen sequence, was designed using SnapGene software. In this construct, the NdeI restriction site was positioned upstream, while the XhoI restriction site was located downstream of the target sequence. The final vector construct is shown in Fig 8.

## Discussion

Bioinformatics analyses conducted in this study indicated that the designed fusion antigen possesses favorable immunological characteristics, supporting its potential application in ELISA‑based diagnostic assays for human strongyloidiasis.

For the construction of the fusion antigen, four protein sequences SsIR, L3NieAg.01, Ss3a, and Ss1a were selected based on their reported antigenic properties and relevance in previous studies [11,12]. SsIR is one of the major excretory‑secretory (ES) proteins expressed during both the invasive larval and adult stages of *S. stercoralis*. It is secreted through the parasite’s ES system and plays a significant role in modulating host immunity [29]. Earlier investigations have shown that SsIR can impair the function of dendritic cells and macrophages, leading to reduced antigen presentation to T lymphocytes and weakening downstream immune activation. This immunomodulatory effect contributes to parasite survival by enabling evasion of host immune defenses [30]. L3NieAg.01, another ES protein expressed by adult parasites, is known to induce robust immune responses. In addition to its involvement in humoral immunity, recent evidence suggests that this antigen may promote parasite attachment to intestinal epithelial cells, potentially supporting tissue colonization and persistent infection [12,31]. Ss1a and Ss3a are ES antigens secreted during the invasive larval stage and are also recognized for their immunogenicity. These proteins stimulate host immune responses and are considered valuable targets for diagnostic and vaccine development [11].

To construct the fusion antigen, selected regions from the four target antigens were assembled into a single continuous polypeptide sequence. The fragments were connected using the EAAAK linker (Fig 4). This linker forms a rigid α‑helical structure that preserves adequate spatial separation between the fused sequences, minimizes steric interference, and enhances the accessibility of epitopes for antibody binding [32]. The EAAAK linker has been widely applied in recombinant multi‑epitope antigen design. For example, it has been used successfully to connect epitopes from five tegument proteins in the development of a multi‑epitope antigen for human cytomegalovirus (*HCMV*) detection [32].

The design of fusion antigens using bioinformatics approaches can significantly enhance the accuracy of serological diagnostic methods. By integrating multiple antigenic sequences from the target pathogen and focusing on regions with high immunogenic potential, such constructs allow for broader antibody recognition while minimizing the risk of false-positive results due to cross-reactivity [33].

Although the proposed construct contains four antigenic components (compared with one or two components in previously reported systems), this design was implemented intentionally. By integrating immunodominant regions derived from multiple antigens, the fusion construct was designed to provide broader epitope coverage while simultaneously eliminating sequence regions associated with potential cross‑reactivity. Therefore, the increased complexity of this construct reflects a rational design strategy aimed at achieving an optimal balance between sensitivity and specificity.

The predicted cross‑reactivity profiles of the full‑length antigens, their selected truncated regions, and the final fusion antigen were evaluated using BLASTp against proteins from several parasitic pathogens that are commonly associated with serological cross‑reactions. The results indicated that the full‑length antigens exhibited varying degrees of sequence similarity with proteins from other parasites. Among them, SsIR showed the highest potential for cross‑reactivity, while L3NieAg.01 also demonstrated predicted similarities with several parasitic proteins. In contrast, Ss3a showed no detectable similarity with any of the evaluated parasites, suggesting a potentially higher level of specificity. Ss1a exhibited only limited sequence similarity with a few parasitic organisms. Importantly, none of the selected truncated fragments used for the construction of the fusion antigen showed detectable similarity to the evaluated parasitic pathogens in the BLASTp analysis. Consequently, the final fusion antigen also showed no predicted cross‑reactivity with any of the examined parasites (Table 2).

Assessing the potential for cross‑reactivity represents a critical step in the rational design of fusion antigens, as the removal of sequence regions that may interact with antibodies generated against unrelated pathogens can significantly improve the specificity of serological assays [9,32].

However, it should be noted that BLASTp‑based analyses provide only an initial level of screening. Sequence similarity alone cannot fully capture the complexity of epitope–antibody interactions. In some cases, antibody cross‑reactivity may occur even when overall sequence similarity is relatively low. Moreover, sequence‑based comparison methods are unable to detect structural mimicry or physicochemical similarities between epitopes. Therefore, the in silico analyses performed in this study should be considered a preliminary assessment of antigen specificity. Experimental validation using sera from patients with other parasitic infections will be necessary to definitively evaluate the diagnostic specificity of the antigen and to confirm the absence of cross‑reactivity under real diagnostic conditions.

These findings are consistent with the study by Omidian et al., who developed a fusion antigen (SsIR–Ss1a) for the serological detection of strongyloidiasis. By aligning the target antigenic sequences with those of other infectious and parasitic agents, the researchers identified immunodominant regions that lacked cross‑reactivity. In their analysis, only three of the 52 serum samples from patients with other parasitic and infectious diseases showed cross‑reactive responses. The SsIR–Ss1a antigen demonstrated a sensitivity of 93.94% and a specificity of 97.22%, indicating strong diagnostic performance for strongyloidiasis [9].

Multi‑epitope and fusion antigens have been widely proposed as effective strategies for improving the specificity of serological assays. However, inappropriate or non‑specific selection of target sequences can reduce diagnostic accuracy and lead to false‑positive results. For example, a study evaluating a chimeric protein (OvCB_OvAEP_OvCF) for the diagnosis of opisthorchiasis reported strong IgG reactivity and achieved 100% sensitivity. However, cross‑reactivity with sera from patients infected with other helminths and protozoa reduced the specificity to 78.4% [34]. Similarly, another study assessed a multi‑epitope antigen for the diagnosis of visceral leishmaniasis using ELISA and Western blot assays. Although the antigen showed high sensitivity (93.1%), its specificity for correctly identifying negative cases was only 77.4%, indicating the need for further optimization [35].

The high diagnostic performance of assays based on NIE and NIE/SsIR antigens has been well documented, with most studies reporting sensitivities and specificities exceeding 90% [36]. Nevertheless, even these widely used diagnostic systems have demonstrated measurable cross‑reactivity. These observations suggest that, despite their strong overall diagnostic performance, the specificity of the currently used reference antigens may still be affected by shared antigenic determinants among phylogenetically related parasites. Furthermore, the number and diversity of heterologous serum samples included in cross‑reactivity panels can also influence the reported specificity values, as the use of broader and more diverse serum panels increases the likelihood of detecting potential cross‑reactive responses [37].

The antigenicity of the full‑length antigens, the selected truncated regions used in this study, and the final fusion construct was evaluated using the VaxiJen server with a threshold of 0.5. As shown in Table 3, all analyzed sequences were predicted to be antigenic. Among the full‑length antigens, SsIR exhibited the highest antigenicity score (0.8672), followed by Ss3a (0.8616), L3NieAg.01 (0.6152), and Ss1a (0.5116). Notably, the selected fragment of SsIR (residues 1–79) showed the highest predicted antigenicity score (0.9873). The selected fragments derived from L3NieAg.01, Ss3a, and Ss1a also retained antigenicity scores above the defined threshold. Furthermore, the designed fusion antigen obtained a VaxiJen score of 0.6949, indicating that the overall antigenic potential was preserved after integrating the selected fragments into a single construct.

It should be noted that the removal of certain sequence regions from individual antigens performed to eliminate segments with potential cross‑reactivity identified through BLASTp analysis may partially reduce the intrinsic antigenic strength of those proteins. In this context, integrating multiple antigenic components derived from the target pathogen into a single fusion construct represents a rational strategy to compensate for this potential reduction. Incorporating several epitope‑rich regions originating from different antigens within a single recombinant construct may broaden the range of antibody recognition across heterogeneous host immune responses and thereby potentially enhance the overall sensitivity of serological diagnostic assays [38,39].

In a related study, the identification of linear B-cell epitopes within the SjSAP4 protein sequence and their diagnostic potential for Schistosoma japonicum infection responsible for schistosomiasis was evaluated. Peptides derived from the SjSAP4 protein were expressed as fusion proteins with a GST tag and subsequently assessed using ELISA. The results demonstrated that a dual-peptide ELISA combining SjSAP4 and SjSP-13V2 peptides achieved a sensitivity of 84% and specificity of 100%, outperforming single-peptide ELISA formats. These findings underscore the diagnostic advantages of combining antigenic epitopes from different proteins to develop more effective tools for detecting schistosomiasis infections [40].

Another study compared the diagnostic performance of individual proteins BP26, Omp25, and Omp31 with that of a multi-epitope fusion protein for the serological detection of brucellosis. The multi-epitope construct demonstrated superior performance, with a positive predictive value (PPV) of 100% and a negative predictive value (NPV) of 98.41%, indicating its high accuracy in differentiating between positive and negative sera compared to single-antigen assays [41].

The physicochemical properties of the designed fusion antigen were assessed using ProtParam, focusing on instability and aliphatic indices. These properties are essential for recombinant protein expression. The instability index was calculated as 39.68, below the threshold of 40, indicating likely stability under physiological conditions [42]. The aliphatic index was 57.83, reflecting the proportion of aliphatic side chains (alanine, valine, isoleucine, and leucine) and suggesting good thermostability [43]. SolPro was used to predict solubility, yielding a probability score of 0.81. This result indicates that the fusion antigen is likely to be soluble, which is important for selecting an expression system and designing extraction and purification strategies [44].

The 3D structural validation of the selected model showed that approximately 90.2% of the predicted amino acid residues were located within the favored regions of the Ramachandran plot, while only a small fraction appeared in disallowed region. The aim of the three‑dimensional modeling in the present study was not to determine the exact atomic‑level structure of the fusion protein, but rather to provide a topological approximation of the spatial arrangement of the selected epitopes. This level of modeling is sufficient for the diagnostic purpose of the study, which focuses on the design of the fusion construct and the evaluation of epitope accessibility, and it can effectively guide subsequent experimental steps, particularly during protein extraction and purification.Since the structural model suggests that certain epitopes may be partially buried within the protein core, such information supports the use of denaturing purification conditions to ensure exposure of these regions. Accordingly, in the experimental phase, it is recommended that the fusion antigen be denatured using 8 M urea and subsequently immobilized in a linear conformation onto the ELISA plate surface. Under these conditions, the proper or improper folding of the protein does not play a decisive role in antigen performance in the ELISA assay. Therefore, the three‑dimensional model is primarily intended to guide the optimization of extraction and purification procedures, rather than to define the precise structural conformation of the fusion protein [32].

Conformational B‑cell epitopes in the final 3D model were predicted using the ElliPro tool. This analysis identified ten discontinuous epitopes. However, it should be noted that the fusion antigen was constructed by linking four distinct sequences with linkers; therefore, the native conformational epitopes present in the parental antigens may not be fully preserved in the chimeric structure. For this reason, the primary focus of the construct design was on linear B‑cell epitopes. Nevertheless, conformational epitope prediction was performed to assess the potential formation of new structural patches within the fusion construct and to provide a more comprehensive overview of its antigenic features [32].

To optimize the expression of the recombinant protein and mitigate challenges associated with codon usage bias, the sequence was optimized. This process involved evaluating several key parameters: CAI, FOP, GC content, and the presence of *cis*-acting regulatory elements, and repetitive sequences.

Following optimization, the CAI reached 0.92 (Fig 7a) [32,45]. This high value indicates strong compatibility between the optimized codons and the host organism’s translation machinery, strongly suggesting enhanced translational performance, as values closer to 1.0 signify preferential use of host-favored codons [32,45]. The FOP analysis showed that the original sequence had a lower proportion of preferred codons (Fig 7b) [32,45]. A higher FOP, achieved in the optimized sequence, implies a greater abundance of codons efficiently recognized by host ribosomes, which can significantly improve protein yield [32,45]. The GC content of the optimized construct was successfully adjusted to 47% (Fig 7c) [32,45]. This value falls within the optimal range (30–70%), which is critical for DNA duplex stability and directly impacts transcription and translation efficiency [32,45]. Crucially, the initial sequence contained one *cis*-regulatory element and six repetitive sequences, elements known to negatively affect expression. These problematic elements were effectively removed during optimization (Table 6), minimizing the risk of premature translation termination and maximizing the expression potential of the target antigen.

The mFold server predicted an mRNA minimum free energy (MFE) of –304.12 kcal/mol after optimization, compared with –391.00 kcal/mol before optimization (Fig 7d). Although lower free energy generally indicates higher structural stability, excessively stable mRNA structures may negatively affect translation efficiency. Highly stable structures can promote the formation of strong hairpin loops that may mask the ribosome‑binding site or impede ribosome progression along the mRNA. Therefore, the reduced structural stability observed after optimization may help decrease rigid secondary structures and improve ribosomal accessibility during translation [46].

The recombinant pET23a(+) vector carrying the fusion antigen was designed using SnapGene software. This vector contains a C‑terminal histidine tag located within the multiple cloning site (Fig 8), which facilitates the purification of the recombinant protein. The choice of affinity tags is an important consideration in diagnostic applications because certain tags may contribute to cross‑reactivity in detection assays. For instance, commonly used expression vectors such as pET32a and pGEX4T1 contain thioredoxin (trx) and glutathione S‑transferase (GST) tags, respectively. These tags share notable sequence homology with several parasitic proteins, which may increase the likelihood of cross‑reactive signals. Moreover, if these tags are not removed after protein expression and remain fused to the antigen, they may act as additional epitopes and further contribute to cross‑reactivity. Such cross‑reactivity could potentially reduce the specificity and reliability of serological tests for the diagnosis of Strongyloides infection [32].

## Conclusion

In this study, we utilized bioinformatics tools to design a novel fusion antigen by selecting regions with high immunogenic potential. This approach was intended to improve antibody recognition while minimizing the risk of false-positive results arising from cross-reactivity. In silico analyses demonstrated that the designed fusion construct exhibits promising immunological features, supporting its potential application in ELISA-based diagnostic assays for human strongyloidiasis. However, a major limitation of this study is the lack of experimental validation. All current findings are only based on computational predictions. Therefore, further experimental studies are crucial to evaluate the construct’s diagnostic accuracy, specificity, and feasibility for clinical implementation.

## Supporting information

S1 File Supplementary data. (XLSX)

## Abbreviations

3D: Three-dimensional
CAI: Codon adaptation index
ELISA: Enzyme-linked immunosorbent assay
ES: Excretory-secretory
FOP: Frequency of optimal codons
GST: Glutathione S-transferase
*HCMV*: *Human cytomegalovirus*
*HTLV-1*: *Human T-lymphotropic virus type 1*
MCS: Multiple cloning site
MFE: Minimal free energy
Mw: Molecular weight
NPV: Negative predictive value
NTD: Neglected tropical diseases
pI: Theoretical isoelectric point
PPV: Positive predictive value
TBM: Template-Based Modeling
trx: Thioredoxin
WHO: World Health Organization.

## References

[1] Al-Jawabreh R, Anderson R, Atkinson LE, Bickford-Smith J, Bradbury RS, Breloer M, et al. Strongyloides questions-a research agenda for the future. Philos Trans R Soc Lond B Biol Sci. 2024;379(1894):20230004. doi: 10.1098/rstb.2023.0004 38008122 PMC10676812

[2] Czeresnia JM, Weiss LM. Strongyloides stercoralis. Lung. 2022;200(2):141–8. doi: 10.1007/s00408-022-00528-z 35396957 PMC8994069

[3] Chan AHE, Thaenkham U. From past to present: opportunities and trends in the molecular detection and diagnosis of Strongyloides stercoralis. Parasit Vectors. 2023;16(1):123. doi: 10.1186/s13071-023-05763-8 37041645 PMC10088203

[4] Vasquez-Rios G, Pineda-Reyes R, Pineda-Reyes J, Marin R, Ruiz EF, Terashima A. Strongyloides stercoralis hyperinfection syndrome: a deeper understanding of a neglected disease. Journal of Parasitic Diseases. 2019;43:167–75. 31263320 10.1007/s12639-019-01090-x PMC6570730

[5] Karanam L SK, Basavraj GK, Papireddy CKR. Strongyloides stercoralis Hyper infection Syndrome. Indian J Surg. 2021;83(Suppl 3):582–6. doi: 10.1007/s12262-020-02292-x 32419745 PMC7223413

[6] Formenti F, La Marca G, Perandin F, Pajola B, Romano M, Santucci B, et al. A diagnostic study comparing conventional and real-time PCR for Strongyloides stercoralis on urine and on faecal samples. Acta Trop. 2019;190:284–7. doi: 10.1016/j.actatropica.2018.12.001 30521805

[7] Hosseini S, Vázquez-Villegas P, Rito-Palomares M, Martinez-Chapa SO, Hosseini S, Vázquez-Villegas P. Advantages, disadvantages and modifications of conventional ELISA. Enzyme-linked immunosorbent assay (ELISA) from A to Z. 2018. p. 67–115.

[8] Arif S, Akhter M, Khaliq A, Nisa ZU, Khan IH, Akhtar MW. Serodiagnostic evaluation of fusion proteins from multiple antigens of Mycobacterium tuberculosis for active TB. Tuberculosis (Edinb). 2021;127:102053. doi: 10.1016/j.tube.2021.102053 33561630

[9] Omidian M, Mostafavi-Pour Z, Asadi M, Sharifdini M, Nezafat N, Pouryousef A, et al. Design and expression of a chimeric recombinant antigen (SsIR-Ss1a) for the serodiagnosis of human strongyloidiasis: Evaluation of performance, sensitivity, and specificity. PLoS Negl Trop Dis. 2024;18(7):e0012320. doi: 10.1371/journal.pntd.0012320 39008519 PMC11271862

[10] Scarso S, Tamarozzi F, Mazzi C, Degani M, Rizzi E, Tais S, et al. Evaluation of novel recombinant antigen-based (NIE/SsIR) immunochromatographic rapid tests for Strongyloides stercoralis: an accuracy study. Parasit Vectors. 2024;17(1):535. doi: 10.1186/s13071-024-06569-y 39716196 PMC11668118

[11] Arifin N, Yunus MH, Nolan TJ, Lok JB, Noordin R. Identification and Preliminary Evaluation of a Novel Recombinant Protein for Serodiagnosis of Strongyloidiasis. Am J Trop Med Hyg. 2018;98(4):1165–70. doi: 10.4269/ajtmh.17-0697 29436335 PMC5928823

[12] Ramanathan R, Burbelo PD, Groot S, Iadarola MJ, Neva FA, Nutman TB. A luciferase immunoprecipitation systems assay enhances the sensitivity and specificity of diagnosis of Strongyloides stercoralis infection. J Infect Dis. 2008;198(3):444–51. doi: 10.1086/589718 18558872 PMC3379004

[13] Noordin R, Arifin N. Strongyloides stercoralis protein and/or corresponding dna and rna sequences for application in diagnosis. WO2017091059A1 (patent). 2017.

[14] Doytchinova IA, Flower DR. VaxiJen: a server for prediction of protective antigens, tumour antigens and subunit vaccines. BMC Bioinformatics. 2007;8:4. doi: 10.1186/1471-2105-8-4 17207271 PMC1780059

[15] Teufel F, Almagro Armenteros JJ, Johansen AR, Gíslason MH, Pihl SI, Tsirigos KD, et al. SignalP 6.0 predicts all five types of signal peptides using protein language models. Nat Biotechnol. 2022;40(7):1023–5. doi: 10.1038/s41587-021-01156-3 34980915 PMC9287161

[16] Saha S, Raghava GPS. BcePred: prediction of continuous B-cell epitopes in antigenic sequences using physico-chemical properties. International conference on artificial immune systems. Springer; 2004.

[17] Sanchez-Trincado JL, Gomez-Perosanz M, Reche PA. Fundamentals and Methods for T- and B-Cell Epitope Prediction. J Immunol Res. 2017;2017:2680160. doi: 10.1155/2017/2680160 29445754 PMC5763123

[18] Saha S, Raghava GPS. Prediction of continuous B-cell epitopes in an antigen using recurrent neural network. Proteins. 2006;65(1):40–8. doi: 10.1002/prot.21078 16894596

[19] Jespersen MC, Peters B, Nielsen M, Marcatili P. BepiPred-2.0: improving sequence-based B-cell epitope prediction using conformational epitopes. Nucleic Acids Res. 2017;45(W1):W24–9. doi: 10.1093/nar/gkx346 28472356 PMC5570230

[20] Walker JM. The proteomics protocols handbook. Springer; 2005.

[21] Cheng J, Randall AZ, Sweredoski MJ, Baldi P. SCRATCH: a protein structure and structural feature prediction server. Nucleic Acids Res. 2005;33(Web Server issue):W72-6. doi: 10.1093/nar/gki396 15980571 PMC1160157

[22] Magnan CN, Baldi P. SSpro/ACCpro 5: almost perfect prediction of protein secondary structure and relative solvent accessibility using profiles, machine learning and structural similarity. Bioinformatics. 2014;30(18):2592–7. doi: 10.1093/bioinformatics/btu352 24860169 PMC4215083

[23] Habib A, Liang Y, Xu X, Zhu N, Xie J. Immunoinformatic Identification of Multiple Epitopes of gp120 Protein of HIV-1 to Enhance the Immune Response against HIV-1 Infection. Int J Mol Sci. 2024;25(4):2432. doi: 10.3390/ijms25042432 38397105 PMC10889372

[24] Seok C, Baek M, Steinegger M, Park H, Lee GR, Won J. Accurate protein structure prediction: what comes next?. Biodesign. 2021;9(3):47–50. doi: 10.34184/kssb.2021.9.3.47

[25] Ko J, Park H, Heo L, Seok C. GalaxyWEB server for protein structure prediction and refinement. Nucleic Acids Res. 2012;40(Web Server issue):W294-7. doi: 10.1093/nar/gks493 22649060 PMC3394311

[26] Laskowski RA, MacArthur MW, Moss DS, Thornton JM. PROCHECK: a program to check the stereochemical quality of protein structures. J Appl Crystallogr. 1993;26(2):283–91. doi: 10.1107/s0021889892009944

[27] Ponomarenko J, Bui H-H, Li W, Fusseder N, Bourne PE, Sette A, et al. ElliPro: a new structure-based tool for the prediction of antibody epitopes. BMC Bioinformatics. 2008;9:514. doi: 10.1186/1471-2105-9-514 19055730 PMC2607291

[28] Zuker M. Mfold web server for nucleic acid folding and hybridization prediction. Nucleic Acids Res. 2003;31(13):3406–15. doi: 10.1093/nar/gkg595 12824337 PMC169194

[29] Abraham D, Hess JA, Mejia R, Nolan TJ, Lok JB, Lustigman S, et al. Immunization with the recombinant antigen Ss-IR induces protective immunity to infection with Strongyloides stercoralis in mice. Vaccine. 2011;29(45):8134–40. doi: 10.1016/j.vaccine.2011.08.030 21856350 PMC3191285

[30] Nutman TB. Human infection with Strongyloides stercoralis and other related Strongyloides species. Parasitology. 2017;144(3):263–73. doi: 10.1017/S0031182016000834 27181117 PMC5563389

[31] Rascoe LN, Price C, Shin SH, McAuliffe I, Priest JW, Handali S. Development of Ss-NIE-1 recombinant antigen based assays for immunodiagnosis of strongyloidiasis. PLoS Negl Trop Dis. 2015;9(4):e0003694. doi: 10.1371/journal.pntd.0003694 25860665 PMC4393093

[32] Asadi M, Ghasemi Y, Nezafat N, Sarkari B, Baneshi M, Mostafavi-Pour Z, et al. Designing a novel multi-epitope antigen for diagnosing human cytomegalovirus infection: An immunoinformatics approach. Biotechnol Appl Biochem. 2025;72(2):469–83. doi: 10.1002/bab.2677 39417400

[33] Huang C, Cao C, Xu Z, Lin Y, Wu J, Weng Q, et al. A blocking ELISA based on virus-like nanoparticles chimerized with an antigenic epitope of ASFV P54 for detecting ASFV antibodies. Sci Rep. 2023;13(1):19928. doi: 10.1038/s41598-023-47068-x 37968284 PMC10651890

[34] Sripa J, Chaiwong T. Multi-epitope protein production and its application in the diagnosis of opisthorchiasis. Parasit Vectors. 2024;17(1):206. doi: 10.1186/s13071-024-06285-7 38715089 PMC11077728

[35] Taherzadeh M, Fouladvand M, Kazemi B. Evaluation of a new multi-epitope sequence of eight known Leishmania infantum antigens for HVL diagnosis by ELISA and Western blot. J Vector Borne Dis. 2021;58(4):289–96. doi: 10.4103/0972-9062.318310 35381816

[36] Lontuo-Fogang R, Nutman TB. Recombinant antigen-based lateral flow tests for the detection of Strongyloides stercoralis infection. PLoS Negl Trop Dis. 2025;19(4):e0013018. doi: 10.1371/journal.pntd.0013018 40198660 PMC12011289

[37] Boonroumkaew P, Sadaow L, Sanpool O, Rodpai R, Thanchomnang T, Phupiewkham W, et al. Effectiveness of Strongyloides Recombinant IgG Immunoreactive Antigen in Detecting IgG and IgG4 Subclass Antibodies for Diagnosis of Human Strongyloidiasis Using Rapid Immunochromatographic Tests. Diagnostics (Basel). 2020;10(9):615. doi: 10.3390/diagnostics10090615 32825495 PMC7555090

[38] Tamarozzi F, Longoni SS, Mazzi C, Pettene S, Montresor A, Mahanty S, et al. Diagnostic accuracy of a novel enzyme-linked immunoassay for the detection of IgG and IgG4 against Strongyloides stercoralis based on the recombinant antigens NIE/SsIR. Parasit Vectors. 2021;14(1):412. doi: 10.1186/s13071-021-04916-x 34407876 PMC8375122

[39] Zhang X, Guo J, Wang L, Li Z, Liu Y, Tian L, et al. Development and evaluation of multi-epitope protein p72 (MeP72) for the serodiagnosis of African swine fever. Acta Virol. 2021;65(3):273–8. doi: 10.4149/av_2021_304 34565155

[40] Mu Y, Gordon CA, Olveda RM, Ross AG, Olveda DU, Marsh JM, et al. Identification of a linear B-cell epitope on the Schistosoma japonicum saposin protein, SjSAP4: Potential as a component of a multi-epitope diagnostic assay. PLoS Negl Trop Dis. 2022;16(7):e0010619. doi: 10.1371/journal.pntd.0010619 35816547 PMC9302751

[41] Yin D, Bai Q, Zhang J, Xu K, Li J. A novel recombinant multiepitope protein candidate for the diagnosis of brucellosis: A pilot study. J Microbiol Methods. 2020;174:105964. doi: 10.1016/j.mimet.2020.105964 32479870

[42] Sarvmeili J, Baghban Kohnehrouz B, Gholizadeh A, Shanehbandi D, Ofoghi H. Immunoinformatics design of a structural proteins driven multi-epitope candidate vaccine against different SARS-CoV-2 variants based on fynomer. Sci Rep. 2024;14(1):10297. doi: 10.1038/s41598-024-61025-2 38704475 PMC11069592

[43] Zhu F, Tan C, Li C, Ma S, Wen H, Yang H, et al. Design of a multi-epitope vaccine against six Nocardia species based on reverse vaccinology combined with immunoinformatics. Front Immunol. 2023;14:1100188. doi: 10.3389/fimmu.2023.1100188 36845087 PMC9952739

[44] Motamedi H, Alvandi A, Fathollahi M, Ari MM, Moradi S, Moradi J, et al. In silico designing and immunoinformatics analysis of a novel peptide vaccine against metallo-beta-lactamase (VIM and IMP) variants. PLoS One. 2023;18(7):e0275237. doi: 10.1371/journal.pone.0275237 37471423 PMC10358925

[45] Mohammadi Y, Nezafat N, Negahdaripour M, Eskandari S, Zamani M. In silico design and evaluation of a novel mRNA vaccine against BK virus: a reverse vaccinology approach. Immunol Res. 2023;71(3):422–41. doi: 10.1007/s12026-022-09351-3 36580228 PMC9797904

[46] Asadi M, Soltani-Fard E, Vosough P, Hajighahramani N, Savardashtaki A, Nezafat N, et al. Designing a self-assembled peptide nano-vaccine against Staphylococcus aureus: An in silico approach. BioNanoScience. 2024:1–16.

